# Automated gigapixel circumferential surface microscopy of the prostate

**DOI:** 10.1038/s41598-019-56939-1

**Published:** 2020-01-10

**Authors:** Samuel Luethy, David B. Tulman, J. Quincy Brown

**Affiliations:** 1 Department of Biomedical Engineering, Tulane University, New Orleans, LA 70118 USA; 2 Bioinnovation Program, Tulane University, New Orleans, LA 70118 USA

**Keywords:** Cancer imaging, Prostate cancer, Surgical oncology, Microscopy

## Abstract

Positive surgical margins, or cancer cells found at the boundary of an excised tumor mass, are a significant problem in the management of many cancers resulting in worsened patient outcomes. The problem is exacerbated in organ sites such as the prostate, where unnecessarily wide local excisions can result in significant deterioration of post-operative quality of life due to collateral damage to neighboring structures. Yet, at the same time, incomplete tumor removal results in worsened prognosis and need for additional interventions. Here, we report the design and development of a rapid and completely automated system for intraoperative gigapixel *ex vivo* microscopy of the circumferential surgical prostate margin within intra-operative timeframes, called the Automated Prostate Positioning System (APPS). The APPS leverages the rotational geometry of the prostate and high speed structured illumination microscopy (SIM) to generate continuous gigapixel panoramas of the fresh intact prostate circumference, including areas of the prostate adjacent to the neurovascular bundles, the rectum, and the bladder wall. Our previous work using SIM and a manual prostate handling method demonstrated the promise of the imaging technique for accurate detection of positive surgical margins. Our work here advances the technology toward clinical adoption, by demonstrating 10% greater tissue surface coverage fraction, 1.6× faster imaging throughput, and reduced number of required operator steps, compared to our prior approach. The APPS may be operated by a single person in the operating room suite within intraoperative time limits, while simultaneously delivering nearly two orders of magnitude higher tissue surface coverage than destructive and labor-intensive frozen section analysis techniques.

## Introduction

Prostate cancer will affect over 174,000 men in the United States in 2019–31,620 of which will die – making prostate cancer the second deadliest form of the disease in males behind lung cancer^1^. Several therapies exist for the treatment of prostate cancer including: external beam radiation, radioactive seed implants (brachytherapy), hormonal therapy, chemotherapy, and surgery. Surgery is a common front-line curative approach to non-metastatic cancer in many solid organs, including the prostate. Prostate cancer surgery, or radical prostatectomy, involves the total removal of the prostate gland including seminal vesicles and ductus deferens, along with surrounding tissue as needed to achieve local control of cancer spread beyond the prostate borders. The objective of successful surgery is to remove the whole prostate organ and any tumor-involved adjacent tissues, such that the prostate cancer is completely contained within the resection specimen, and no tumor cells are found at the border of the specimen. Absence of tumor cells at the inked surgical margin is called a negative surgical margin (NSM), whereas a positive surgical margin (PSM) indicates presence of tumor cells at the inked boundary of the resection specimen. Positive surgical margins are a frequent occurrence (11–38% of all radical prostatectomy surgeries regardless of clinical stage, and exceeding 50% in pT3 and pT4 tumors)^2,3^, and are associated with increased chance of biochemical and local tumor recurrence and are a poor independent prognostic indicator^2,4,5^. Significant PSM’s are also a trigger for additional harmful treatments including adjuvant radiotherapy. However, there is a tension in radical prostatectomy to be sufficiently radical to remove all of the tumor for oncological safety, yet to also maximize preservation of the neurovascular tissues and organs adjacent to the prostate to minimize co-morbidities and decreased quality-of-life. Thus, there is a need for useful methods to identify PSMs intra-operatively to serve as a safety-net for nerve-sparing radical prostatectomies, enabling optimal tissue preservation and oncological safety.

The only currently available solution for intra-operative correction of PSMs is targeted frozen section analysis (targeted FSA), in which small pieces of tissue from areas of concern identified by the surgeon are removed and sent for freezing, cryosectioning, mounting onto glass slides, staining, and analysis by a pathologist. Due to the labor and time-intensiveness of FSA, however, this approach is 1) limited to small areas, and 2) dependent on whether the tissue collected by the surgeon was accurately sampled from the tumor-involved area, which have likely contributed to the low sensitivity of the technique^6–8^. Newer highly-intensive FSA techniques, including neurovascular structure-adjacent frozen-section examination (NeuroSAFE), have been developed for intraoperative evaluation of the prostate surgical margin in the lateral and postero-lateral aspects. The NeuroSAFE trials demonstrate that intensive intraoperative assessment of the surgical margin is useful to convert positive surgical margins to negative status intraoperatively, and that surgeons are willing to wait up to an hour to obtain this benefit. However, these approaches are still limited by labor-intensiveness, requiring multiple trained personnel in the operating room and pathology suite to complete, and sub-optimal surface sampling due to the cryosectioning approach^9^.

Our group has advanced structured illumination microscopy (SIM) for the *ex vivo* imaging of entire tumor surgical margins as an alternative to intensive FSA, with the goal of intra-operatively capturing gigapixel histologic panoramas of the resected tissue surface for use in the identification and correction of PSMs during the primary operation^10,11^. SIM allows for the rapid and non-destructive imaging of the circumferential margin of radical prostatectomy specimens without the time intensive embedding and cutting steps^10^. Previous works have demonstrated the diagnostic accuracy of SIM images and the ability of SIM to define features similar to traditional pathology methods^12–14^. Despite the advantages offered by SIM, the previous method for intra-operative imaging required manual re-positioning of the prostate organ on the microscope stage to image adjacent surfaces, and did not always allow for continuous imaging of the circumferential surface depending on the shape of the prostate^10^. The consequences of the need for manual handling included prolonged intra-operative time required to carefully re-position and orient the prostate, and the potential for missed areas between adjacent surfaces.

In this work we analyze the problem of whole-organ or whole-specimen surface imaging using epi-illumination fluorescence imaging, with human prostate as the specific example. We first describe a theoretical analysis of tissue surface image coverage using our previously established method, and a newly developed method described here. We measure the surface coverage of whole prostate SIM imaging using our previously established manual sample handling method, by comparing scanned areas to theoretical computations and surface area measured using 3D structured light scanning of the prostate. We then describe a new method and device for fully-automated whole organ imaging using a novel sample handler leveraging rotational geometry of the specimen, and compare the performance of the system to our previous methods in terms of surface coverage, and total sample handling and imaging time. The developments and findings from this work further advance the utility of SIM for the intra-operative detection of surgical margins, by demonstrating a practical automated prostate positioning system (APPS) that enables automated hands-free gigapixel prostate imaging with complete circumferential surface coverage within intra-operative timeframes.

## Theoretical Analysis

**Figure 1:**
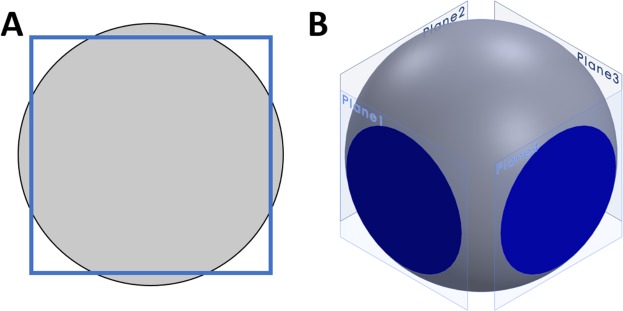
Theoretical image surface coverage of a compressible sphere, simulating tissue resting against a glass slide. 2D (**A**) and 3D (**B**) theoretical representations of prostate surface coverage of SIM imaging using manual rotation of the specimen to image four sides. The 2D surface area imaged with SIM is represented by the intersection of a compressible sphere with four planes.

The method for tumor margin imaging pursued by our group relies on imaging the surface of the excised tissue using an inverted epi-illumination optical sectioning microscope, specifically in our case a structured illumination microscope, described previously^10,12,15,16^. To image the irregular surface of large tumor resections such as a radical prostatectomy specimen, we leverage the compressibility of the fresh specimen *under its own weight* to create a flat tissue surface against a large glass slide, through which an imaging system views the tissue surface. It is important to note that at no time does our method apply any compression to the tissue to deform it against the glass slide – we have found that the weight of large resection specimens provides adequate pressure to flatten the tissue surface for imaging with our system. To analyze theoretically the maximum surface coverage achievable using such a method, it is instructive to consider the surface area coverage that can be achieved using our previously reported imaging method of manually rotating the tissue 90° about the urethral axis in-between image mosaic acquisitions. Consider a cross-section of the prostate as a compressible circle and the area of tissue in contact with the microscope slide as the intersection between this compressible circle and a square inscribed around the circle (Fig. 1A). When expanded to three dimensions, the circle becomes a sphere and the square becomes four planes. The area of tissue in contact with the slide is now the 2D area where the sphere and planes overlap (Fig. 1B).

**Figure 2:**
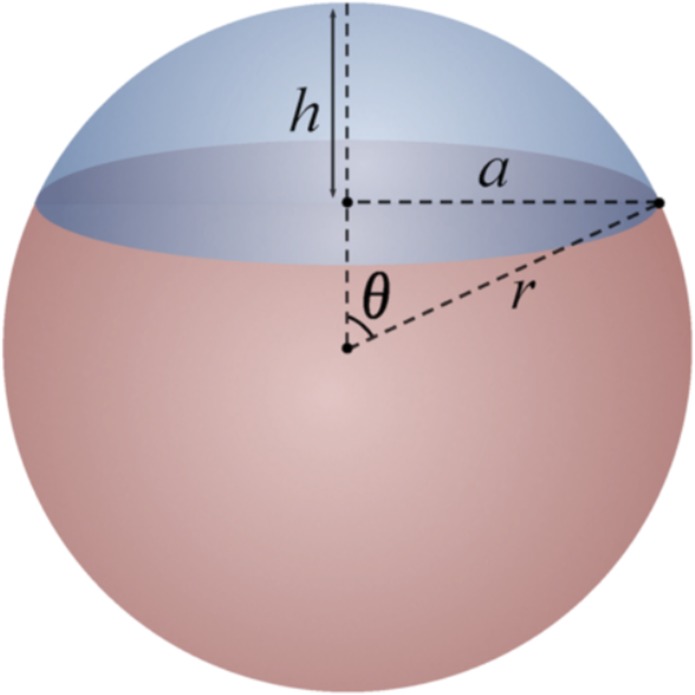
Diagram of spherical cap. The area of intersect between the compressible sphere and the microscope slide is the circle with radius, *a*.

To calculate the surface area of the intersection between the compressible sphere and the four planes, we modify the equation for the area of the base of a spherical cap (Fig. 2, equation 3.2)

The equation for the radius, *a*, of a spherical cap, is given by:

1 $$a=\sqrt{2rh-{h}^{2}}$$

We consider a compressible sphere; therefore, *h* will vary depending on the compressibility of the tissue. The compressibility of the tissue can be expressed in terms of the radius of the sphere and a compressibility coefficient, *c*. Therefore, *h* becomes *rc*, or the radius of the sphere multiplied by the compressibility coefficient. Equation 1 then becomes:

2 $$a=\sqrt{2{r}^{2}c-{r}^{2}{c}^{2}}$$

Therefore, the area *A* of the base of the spherical cap, or the area of intersection between the compressible sphere and the microscope slide is given by:

3 $$A=\pi (2{r}^{2}c-{r}^{2}{c}^{2})$$

The theoretical surface image coverage achieved as a percentage of the total surface area of the prostate, *S*_*I*_, can be determined by dividing this area of intersect by the total surface area of the sphere and multiplying by four (to represent the 4 surface aspects):

4 $${S}_{I}=4(\frac{\pi (2{r}^{2}c-{r}^{2}{c}^{2})}{4\pi {r}^{2}})=2c-{c}^{2}$$

**Figure 3:**
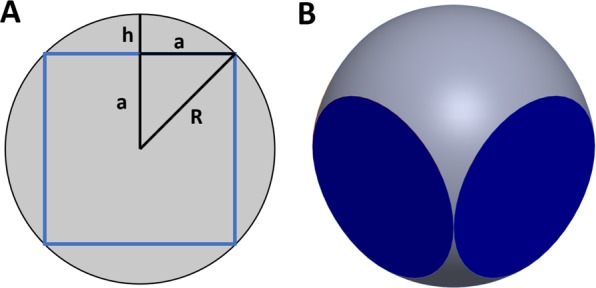
Theoretical model of the imageable surface area at maximum compressibility of a spherical prostate. It is useful to show this diagram in 2D (**A**) and then use the calculated relationship between h and R to determine the compressibility of the tissue in terms of R. This value can then be used in equation 3.5 to calculate the maximum theoretical surface area coverage.

Equation 4 shows that the percent surface image coverage of a prostate specimen approximated by a compressible sphere depends only on the compressibility of the tissue. The prostate, being a relatively dense gland, has an estimated compressibility of 20% of the radius (based on measurements of prostate dimensions and resulting images of the flattened tissue areas in 31 patients, data not shown). Using 20% as *c* in Eq. 4 returns 36% as the total theoretical surface area coverage of four aspects of the prostate using the method thus described. Let us consider the maximum compressibility of the prostate in this scenario to obtain an upper bound on imageable surface area using this method. If we assume that the maximum percent compressibility of the prostate is the percentage at which the four planes that represent the flattened and imaged areas begin to touch, this compressibility can be calculated to be derived in terms of radius, R (Fig. 3, Equation 3.6).

5 $$\begin{array}{ccc}{R}^{2} & = & 2{a}^{2}\\ a & = & \frac{R}{\sqrt{2}}=R-h\\ h & = & R(1-\frac{1}{\sqrt{2}})= \sim 0.29R\end{array}$$

Therefore, the percent compressibility, *c*, that would result in the intersection of the four planes is approximately 29%. Using this value in Eq. 4, we obtain exactly 50% as the upper theoretical limit of image surface coverage achieved by rotating the prostate 90° and imaging the entire surface in contact with the slide, assuming a highly compressible tissue. Our experimental data suggest that the compressibility of the prostate under its own weight is closer to 20%. The prostate could likely be compressed additionally to achieve the 29% deformation needed to achieve 50% surface coverage, however, this could introduce distortions to the images of the tissue, and one can see from Fig. 2 that this method still misses aspects of the prostate circumference, which is the most important area for detection of residual disease when using a system such as this as a “safety-net” for nerve-sparing radical prostatectomy. Given this, the maximum upper bound on surface coverage for our original method is likely closer to 36%.

**Figure 4:**
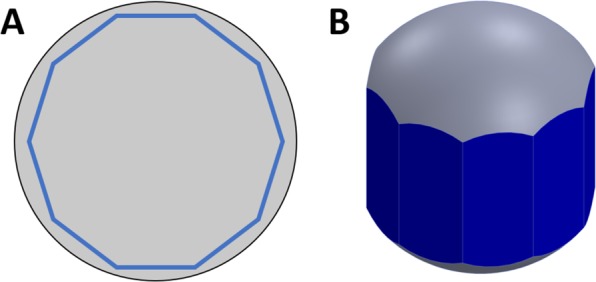
Model of proposed surface area coverage. 2D (**A**) and 3D (**B**) representations of resultant surface coverage of new SIM tissue handling procedure.

We therefore sought to develop a new method to solve 2 problems with the original method: 1) the need for manual rotation, balancing, and determination of image size of the prostate in-between imaging each surface, and 2) the need to increase the circumferential surface image coverage of the prostate. Given that the urethra passes through the longitudinal axis of the prostate (from apex to base), we decided to leverage the rotational geometry to enable contiguous imaging of the prostate circumference. Specifically, we arrived at the notion of mounting the prostate on a spinning rod, and imaging the circumference of the prostate in smaller standard-sized strips extending from apex to base, followed by automated lifting, rotation, and lowering of the prostate in-between each image acquisition. Consider now a circle inscribed in a decagon (Fig. 4A). The decagon represents the field of view of the mosaic images collected for the tissue circumference using the new imaging protocol. Notice that the width of the imaging field for any one prostate orientation has been reduced, such that system covers the full length but only a fraction of the circumference in contact with the slide at any given time. By precisely orienting the prostate over multiple rotations and imaging a predefined tissue width based on the tissue circumference at each rotation, we ensure that no aspect of the circumference goes unimaged, increasing imaged surface area coverage of the margin (Fig. 4B). If the number of imaged surfaces is 10, then the theoretical surface image coverage is 48% for a prostate of compressibility of 20%. Therefore, this method has a number of advantages over our previous method. Firstly, it increases theoretical tissue surface coverage from 36% to 48% for the same compressibility of 20%. Secondly, because the rotations and the width of each image strip are standardized, the method can be fully automated, which reduces complexity and imaging time, since there is no need to manually rotate the specimen and determine the imaging boundaries needed to image the full area in contact with the glass slide. Finally, it enables contiguous surface coverage of the entire organ circumference without the need for artificial compression of the prostate, which was not possible with our prior method. In the next section, we describe the development of the automated prostate handling system and a comparison of experimental results to our theoretical analysis.

### Automatic Prostate Positioning System (APPS) design

**Figure 5:**
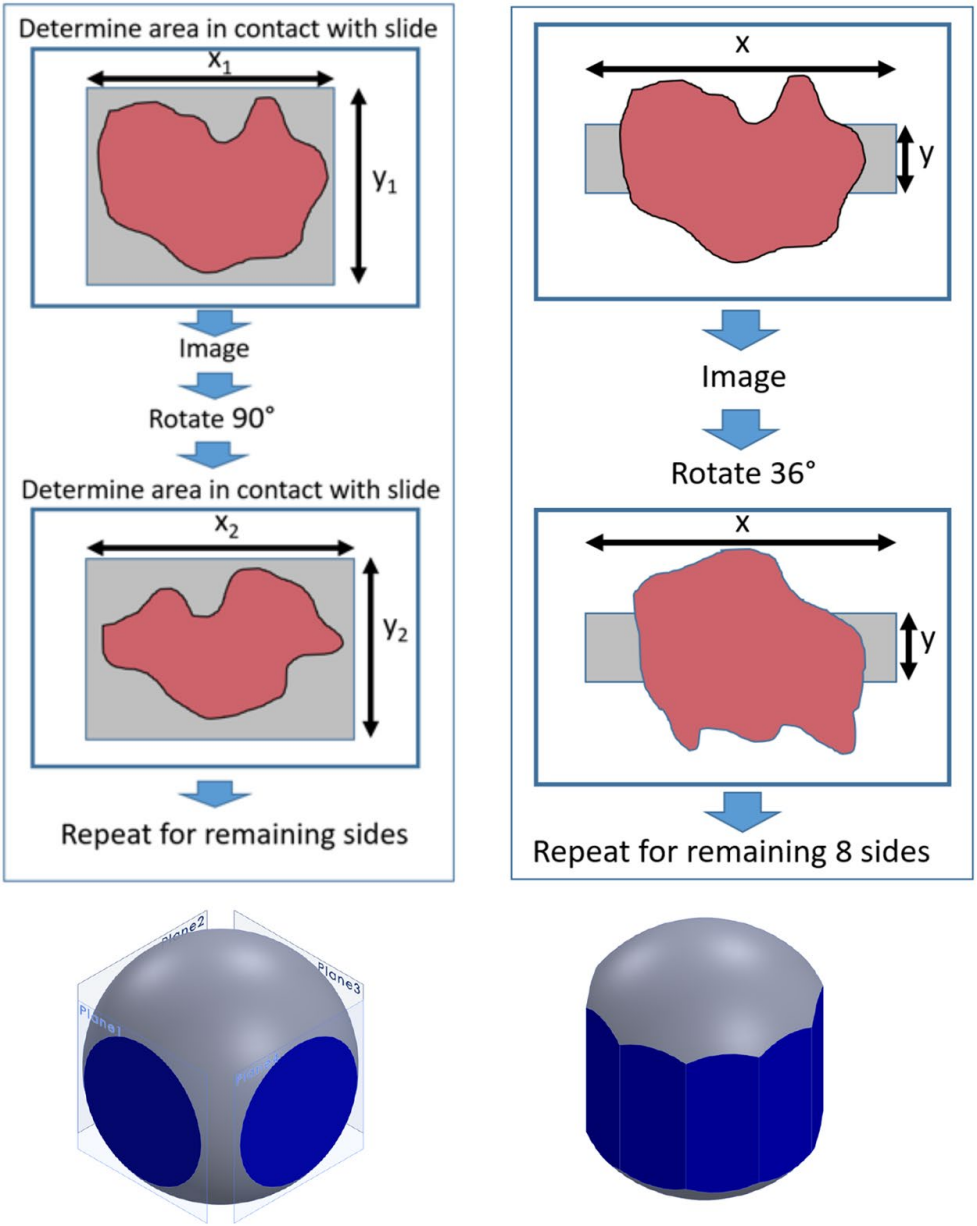
Manual and automatic tissue handling methods. (**A**) In the manual process, in order to capture as much surface area of the prostate as possible when manually rotating the tissue between image captures, the user must manually determine the area of the slide to be scanned between each 90° rotation. (**B**) In the APPS process, by precisely rotating the tissue and imaging the same area of the microscope slide between each rotation, the APPS dramatically reduces the amount of manual input.

**Figure 6:**
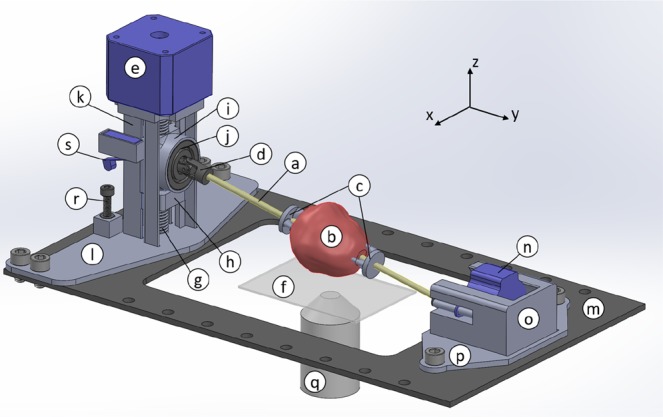
Automated Prostate Positioning System (APPS). Before imaging begins, a wooden dowel rod (**a**) (d = 3 mm) is placed through the prostatic urethra. The prostate (**b**) is held in place on the dowel rod with 3D printed blunt tripod mounts (**c**) that are press-fit onto the rod. The ends of the dowel rod are secured in gimbal joints (**d**) with set screws. Stepper motor 1 (**e**) lowers the prostate tissue onto a 50 × 75 mm glass slide (**f**) which is secured in stage clamps (not pictured). To lower the prostate onto the slide, stepper motor 1 turns a threaded rod (**g**) which engages a nut (not pictured) that is prevented from spinning by a 3D printed housing (**h**). By preventing the screw from spinning, axial motion is produced as the ‘fixed-nut’ component moves up and down in the *z*-direction as the threaded rod rotates about the *z*-axis. On top of the fixed-nut housing rests the free-floating bearing housing (**i**) which the threaded rod does not engaged. The bearing (**j**) inner diameter engages the gimbal joint with a press fit. The threaded rod, fixed-nut housing, and bearing housing all reside in the 3D printed tower (**k**) which is fastened to stepper motor 1 via screws. The tower and internal components are attached to the tower via the mounting plate (**l**) which is attached to the stage (**m**) with screws. A second gimbal joint connects stepper motor 2 (**n)** to the other end of the dowel rod. Stepper motor 2 is responsible for the rotation of the dowel rod and therefor the prostate. Changing heights of the bearing housing result in a changing distance from stepper motor 2 to the base of the tower. This is compensated for by allowing stepper motor 2 to slide in its 3D printed housing (**o**) as the prostate is raised and lowered. The stepper motor 2 housing is attached to the stage via the 3D printed stepper motor 2 housing base plate (**p**). During image acquisition, the stage moves in the *xy*-plane in a serpentine pattern above the objective (**q**) to cover the desired surface area. When the imaging is complete, stepper motor 1 activates, raising the tissue, followed by stepper motor 2, rotating the tissue, followed by stepper motor 1 in the opposite direction, lowering the tissue back onto the slide for the next image acquisition. Prior to the first image being taken, a thumb-screw (**r**) is adjusted such that a microswitch (**s**), which is attached to the floating bearing housing, is triggered as the prostate contacts the slide and rests under its own weight. The microswitch interrupts the lowering of the prostate and begins a new image acquisition.

In consideration of the long-term goal of having an imaging system in the operating room performing intraoperative analysis of surgical margins, it is obligatory that imaging time be reduced as much as possible, while simultaneously maximizing tissue coverage. An additional goal is for the process to be completely automated, such as to minimally interfere with current intra-operative workflows. To this end, we have developed a novel Automatic Prostate Positioning System (APPS) to eliminate the need for human input once the imaging process has begun. This device uses the natural location of the urethra through the long axis of the prostate to conveniently manipulate the prostate rotationally, and stepper motors to lift, rotate, and lower the prostate between image acquisitions. This reduces imaging time because the entire organ circumference is systematically sampled using small precise rotation steps and a pre-defined image strip width, eliminating the need to manually find the outer edge of the prostate in contact with the microscope slide at each rotation (Fig. 5). It also has the added benefit of increasing tissue surface-glass contact over the imaging plane. The device is shown in Fig. 6, and its operation is described in detail in the figure caption. Two additional minor components were fabricated to aid in the use of the APPS. First, custom blunt tripod mounts were fabricated to hold the prostate in place on the dowel rod as it spins. Second, a microscope slide booster was fabricated to raise the height of the imaging plane so that the tissue mounted in the APPS makes full contact with the microscope slide. This slide booster mounts to the stage in the same manner as a microscope slide.

This new device and imaging protocol results in a ‘rolled out’ view of the prostate instead of four separate images, allowing for ease of correlation between excised tissue, images, and patient anatomy. In addition, by increasing the number of aspects taken of the prostate, we increase imaged surface area. An accepted time frame for intra-operative FSA is approximately 20–30 minutes for standard FSA, and circa one hour for intensive FSA such as NeuroSAFE. Therefore, the goal of the APPS is to allow for the imaging of the full circumferential margin in this same timeframe or less.

## Methods

### Imaging instrumentation

The imaging system used in this work was a custom structured illumination microscope (SIM) system described previously^10,12,15^. Briefly, it is constructed around an automated epi-fluorescence microscope platform (RAMM, Applied Scientific Instrumentation), which incorporates a 7 mm/s motorized XY specimen stage and a motorized Z objective positioner. Two different light sources were used over the course of data collection for this report. Earlier samples were illuminated by a 475 nm LED from Thorlabs, and later samples were illuminated with 470 nm laser light from a multimode-fiber-coupled laser engine (LDI-6, 89 North). Excitation light is transmitted through a polarizing beam splitter and imaged onto a liquid crystal on silicon (LCoS) spatial light modulator (SLM, Model 3DM, Forth Dimension Displays). The excitation light is filtered and reflected into the imaging objective (Nikon, Plan Apo 10 × 0.45 NA) by a multiband dichroic mirror, projecting the SLM-generated pattern onto the sample. Fluorescence from the sample is collected by the objective and transmitted through the dichroic mirror and a multiband emission filter. The image is collected by a scientific CMOS camera (Orca Flash 4.0 v2, Hamamatsu) at a full-frame resolution of 2048 × 2048 pixels with a pixel size of 0.645 μm at the sample. Thus, at 10X magnification, the single-frame field-of-view is 1.3 mm × 1.3 mm and the lateral resolution was 1.95 μm (the equivalent of 3 pixel widths). The lateral resolution was confirmed by imaging a 1951 USAF resolution target (2015a USAF, Ready Optics) placed on top of the 1 mm thick glass slide used for imaging. Group 9 Element 1 was clearly resolved, which has a bar width of 0.977 μm and a full pitch width (i.e. the inverse of the spatial frequency of 512 line pairs/mm) of 1.95 μm. Synchronization and control of the LED, SLM, stage, objective, and camera was achieved via custom-written LabVIEW software (National Instruments) and home-built electronic triggering circuits.

### Image acquisition using manual rotation

Intact prostates obtained from robotic radical prostatectomy procedures performed at Tulane University Hospital were transported directly from the operating room to the nearby imaging lab within 10 minutes of excision. Informed consent was obtained from all patients in accordance with a protocol approved by the Tulane Biomedical Institutional Review Board. All methods were carried out in accordance with the approved guidelines and regulations. The prostates were rinsed with PBS to remove excess blood or fluid from the surface, blotted dry with lab tissue, and fully-immersed in a beaker containing 0.5% acridine orange in phosphate buffered saline (PBS) for 30 seconds. Following the staining, the prostates were rinsed in a beaker containing PBS and again blotted dry. Specimens were then directly imaged with SIM, using either the previously-reported manual 90° rotation method^10^, or the new APPS device. For each specimen, the prostate surface was placed on a 75 × 50 × 1 mm glass slide and gentle pressure was applied during initial positioning so that the surface of the prostate adhered to the glass. The slide was then mounted on the stage of the SIM system. No compression was applied to the prostate during imaging. Before imaging, the stage was manually adjusted such that the center of the tissue to be imaged was positioned above the objective. The objective was then moved in the z-direction until the tissue was properly focused. This focal distance was maintained for all images taken of one aspect of the prostate. Then, the user positioned the microscope objective under the outermost southeast corner of the specimen, and then zeroed the scan stage. From this “home” position, the user manually scanned the stage while watching a live video feed of the specimen and noting the position of the stage on the stage controller display, in order to determine the appropriate x-direction and y-direction scan limits needed to cover the entire surface in contact with the slide. Once these limits were determined, the stage was returned to the home position, and the scan dimensions were input into the custom LabVIEW software. Automatic image scanning was then initiated in the software. After completely imaging one entire surface with SIM, the specimen was manually rotated along the urethral axis, and the previously described positioning and imaging procedure (including determining the image scan limits) was repeated three to four more times until the following approximate surfaces of the prostate (posterior, anterior, right lateral, left lateral, and in some cases the base) were imaged within a one-hour timeframe. Most often, only four aspects of the tissue were captured corresponding to the circumferential aspect, and the sample was manually rotated 90° between each acquisition.

Incoherent SIM was performed by projecting a sinusoidal pattern onto the sample, which is phase-shifted by one-third of the grid period between each of three sequential images. From these three patterned images, a single optically-sectioned image was obtained using the square-law detection algorithm described by Neil *et al*.^17^.

6 $${I}_{SIM}=\sqrt{{({x}_{1}-{x}_{2})}^{2}+{({x}_{1}-{x}_{3})}^{2}+{({x}_{2}-{x}_{3})}^{2}}$$

where I_SIM_ is the recovered optically sectioned image, and x_1_, x_2_, and x_3_ are the three sequential patterned images, respectively. Mosaics of the stained tissue were collected using a serpentine scan approach. Each individual frame in the mosaic was first corrected for non-uniform illumination (i.e. flat field correction) by dividing by an intensity-normalized reference image taken of a fluorescent calibration slide (Chroma) or a thin layer of fluorescent dye on a coverslip. Images collected with overlap between frames were stitched using the Grid/Collection Stitching plugin in FIJI. The processed images were re-scaled to restore them to 16-bit grayscale intensity and saved as full resolution TIFF or BigTIFF format. The images were then subsequently converted to multi-resolution tiled pyramidal TIFF or BigTIFF format using nip2 software for use in zoom-and-pan web viewers.

### Image acquisition using the APPS device

**Figure 7:**
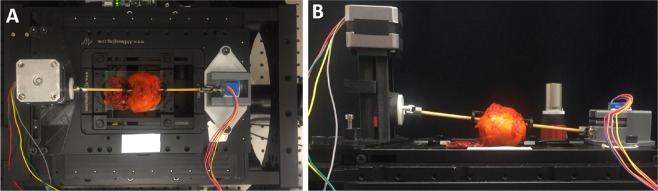
Photographs of a prostate mounted on the APPS system for imaging. (**A**) Top view, (**B**) side view.

Intact prostates obtained from robotic radical prostatectomy procedures were obtained and stained as before. For use with the APPS device, a wooden dowel rod was then placed through the prostatic urethra and secured with custom fabricated plastic tripod mounts. The prostate with dowel rod in place was then mounted in the APPS system, which itself was mounted to the stage of the SIM imaging system (Fig. 7). To reduce human input, the number of aspects or “panel” images taken of the prostate, or the number of times the tissue was rotated, was held constant at 10. Generally, the number of aspects and the diameter of the prostate sample determines the width of each “panel” or strip. A larger diameter prostate will require more frames per panel width to cover the entire circumference in ten rotations, compared to a prostate of smaller diameter. The equation to calculate the width of each panel required to capture the full circumferential margin in ten rotations is simply the arc length of a 36° angle on a prostate with diameter, d:

7 $$Arc\,lengh=\pi d(\frac{C}{360})$$

where C is the central angle of the arc in degrees, again, 36° in this case. The equation becomes:

8 $$Width\,of\,imaging\,panel=\frac{\pi d}{10}$$

Before imaging began, the diameter of the prostate was measured to the nearest millimeter using a ruler (rounding up) to determine the width of each panel according to Eq. 8. The length of each panel was measured to be the straight line distance from the base to the apex of the prostate, also measured using a ruler to the nearest millimeter, rounding up. The calculated length and width dimensions were then input into the custom software, which automatically calculates the number of images needed in the x and y directions for each panel. The size and x-y coordinates of each panel were kept constant for the entire imaging session. Although our system incorporates image-contrast-based autofocus capability, we have found over numerous studies that autofocus is generally not needed for imaging whole prostates, due to the deformability of the fresh prostate resting under its own considerable weight. Therefore, as for the manual method, a single plane of focus was determined for each panel, and was kept constant for each frame in each individual panel. The first image panel was collected, and then APPS system was used to automatically rotate and position the prostate in-between each of the remaining 9 surface panels. The time between the beginning and end of the imaging protocol was recorded, that is, when the first and last images were taken. This figure does not include pre-imaging steps such as staining and mounting in the APPS. Each individual SIM frame computed from 3 sequential images required between 30–75 ms of total SIM image acquisition time, depending on the selected single-frame camera integration time (i.e., 10 ms or 25 ms). Approximately 200 ms was required to move the stage between each frame location. Images were acquired only when the microscope stage was stationary, avoiding any potential issues with motion blur.

Mosaics of SIM images captured of the prostate surface while using the APPS were processed in the same way non-APPS images were. However, rather than displaying each surface as a different image as in our previous work, margin images captured using the APPS were displayed in one continuous strip or ‘rolled out’ view of the entire organ circumference. Imaging always started with the center of the posterior side of the prostate, so that the relative location on the prostate of any particular area of an image could be determined.

### Experimental determination and comparison of prostate tissue surface image coverage

To measure the tissue surface image area coverage and compare to theoretical predictions, the total area of tissue measured by the imaging system was extracted from the resulting images and compared to surface area measured using a 3D scanner. To determine the tissue surface area coverage achieved by SIM imaging, an analysis was performed using a thresholding method. Specifically, grayscale images were converted to binary images to isolate pixels corresponding to “tissue” versus those corresponding to areas outside of the tissue area. All threshold analyses were performed in MATLAB using Otsu’s method to convert from grayscale to binary images^18,19^.

**Figure 8:**
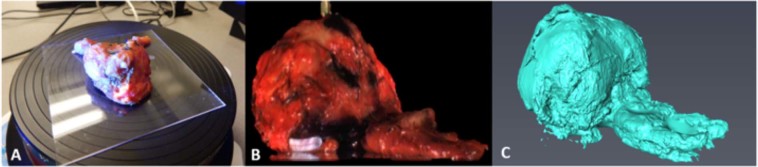
3D scanning workflow. (**A**) Tissue specimens are placed on an automatic 360° turntable, (**B**) scanned using structured light generating a 3D color solid surface image, and (**C**) exported to MeshLab to determine the surface area. Note: Ductus deferens and seminal vesicles are included here for visualization purposes, but were not included in the calculation of SIM image surface area or measurement of 3D scan surface area.

Excised prostates vary greatly in dimension from patient to patient, both in terms of size and shape. Therefore the surface area must be measured for each individual patient, yet the prostate is bulky and irregular, thus surface area approximations such as spherical or ellipsoidal from simple length measurements, may not always be accurate. To obtain the actual surface area of the prostate to compare with SIM images, a structured-light 3D scanner was used. 3D scans of excised prostates were achieved using a DAVID-SLS-2 3D scanner and automatic turntable (Fig. 8).

Finally, to determine the percent surface area coverage obtained using SIM for the two separate prostate handling methods, the total imaged tissue surface area for a particular case was measured and divided by the total tissue surface area as determined by the 3D scan. Seminal vesicles and ductus deferens, which extend from the base of the prostate, are not used in the post-operative diagnosis of circumferential surgical margins using slide-based pathology. For this reason, 3D scans were manually altered to remove ductus deferens and seminal vesicles, and total surface area of SIM images were also calculated without the inclusion of the seminal vesicles or ductus deferens.

## Results

### The APPS enables full-circumference imaging of freshly-excised human prostates

**Figure 9:**
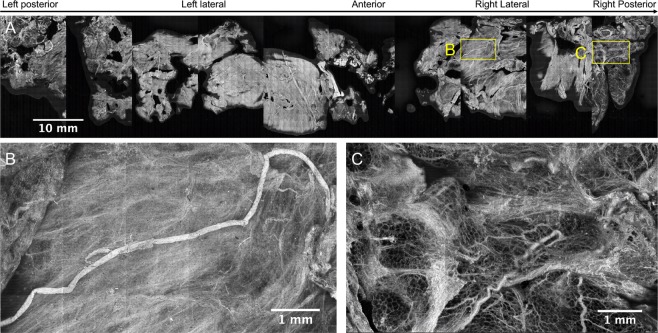
Full circumference microscopic image of the human prostate surface – Case 58. “Rolled-out” view of the circumferential resection margin of case number 58. Approximate anatomical locations are indicated above the image. The image is comprised of 2,700 individual 4.2 megapixel SIM frames giving a total image area = 42 cm^2^, and the area of the image corresponding to tissue = 30.64 cm^2^ at 1.95 μm lateral resolution (0.65 μm pixel resolution).

To date, 5 radical prostatectomy circumferential surgical margins have been imaged using the APPS, 3 of which were used to finalize design requirements and code, and to test device repeatability, and therefore were not fully imaged. Figure 9 shows the corresponding resulting image from the prostate mounted in the APPS shown in Fig. 6 (Case 58). Approximate locations of posterior, left, anterior, and right are indicated above the image. This 10.75 gigapixel image is stitched from 2,700 individual 4.2 megapixel images covering an area of ~42 cm^2^, of which 30.64 cm^2^ corresponds to tissue surface area (determined using the image thresholding method described above). The Nyquist-limited resolution of the image is 1.3–1.94 μm (2–3 pixels at 0.65 μm pixel resolution), and single cell nuclei are readily observed in the image. To get an idea of the scale of this image: if a single cell nucleus of diameter ~10 μm (comprising approximately 185 image pixels) were scaled to the equivalent diameter of a soccer ball, then the image of Fig. 9 would need to be correspondingly scaled to an area equal to ~3,217 regulation soccer pitches, in order to maintain the same image-size-to-feature ratio. Fortunately, characteristic features in the image are much larger than the size of a single cell nucleus, and so it is feasible to visually scan the image within an intraoperative timeframe to locate features of interest.

Figure 9B,C contains examples of typical features observed on the surface of the excised human prostate, based on a previous image atlas that we generated previously. Figure 9B contains a single peripheral nerve bundle with a continuous length of 9.4 mm and width of ~129 μm, against a background of dense periprostatic fascia with scattered microvessels. This snapshot is from the right lateral circumferential surface, where ample neurovascular bundles are expected to be found within and adjacent to the periprostatic fascia, if present on the surgically removed specimen. Conversely, Fig. 9C contains an area of fascia with ample adipose tissue, collagenous connective tissue, and scattered vessels from the posterior or dorsal aspect of the prostate. Markedly fewer nerves are observed in this region compared to the dorsolateral and anterolateral aspects of the prostate, as expected. These images demonstrate the ability to observe the variation in prostatic microanatomy over the entire prostate surface, and are consistent with current understanding of prostate anatomy based on histological and anatomic analysis.

**Figure 10:**
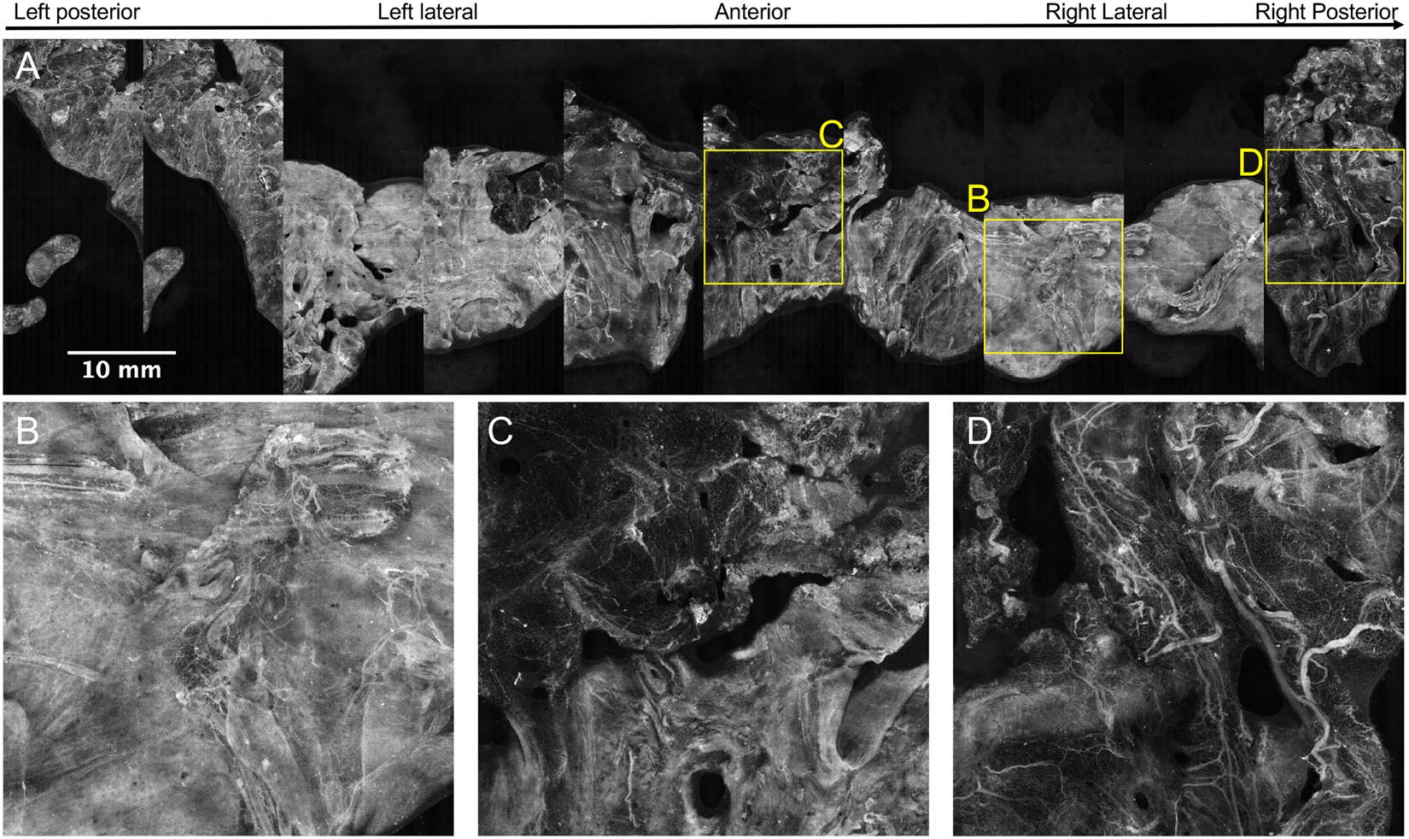
Full circumference microscopic image of the human prostate surface – Case 57. (**A**) This 12.5 gigapixel stitched image is comprised of 2,970 individual 4.2 megapixel SIM frames; the total area of the image corresponding to tissue = 26.63 cm^2^. In (**B**) bright, dense stroma and cords of smooth muscle with an abundance of neurovascular bundles are observed, characteristic of the left and right lateral aspects of the prostate. In (**C**) less bright and dense stroma with a higher abundance of adipose tissue and lower density of neurovascular bundles are observed, characteristic of the anterior aspect of the prostate. In (**D**), dim stroma with abundant adipose tissue with numerous but scattered vessels and nerves.

There are apparent areas of the prostate in Fig. 9 in which the area of imaged tissue is low, specifically at the anterior aspect of the prostate. It is our experience that the anterior surface of the prostate, which is composed of the anterior fibromuscular stroma, can in many cases have a rougher topography after excision than other areas of the prostate. We did not employ autofocus in these studies, but this could be used to increase tissue coverage in areas where tissue surface roughness is high. In contrast, Fig. 10 shows a circumferential image of a prostate where this loss of tissue coverage is not apparent (Case 57). The variation in types of microanatomy over the different zones of the prostate circumference is very clear in this specimen, with the right and left lateral surface (Fig. 10B) being characterized by bright and dense stroma and cords of smooth muscle with the highest abundance of neurovascular bundles; the anterior surface (Fig. 10C) being characterized by slightly less bright and less dense stroma and smooth muscle combined with some adipose tissue and a lower density of neurovascular bundles; and finally the posterior surface (Fig. 10D) being characterized by the dimmest and least dense stroma admixed with abundant adipose tissue and numerous but scattered nerves and vessels (i.e., not the well-organized neurovascular bundle complexes seen on the lateral and anterolateral aspects). Neurovascular bundles are characterized by highly aligned large nerves with associated small vessels, all embedded within a collagenous stroma incorporating adipose tissue which is thought to serve the purpose of supporting and cushioning the neural tissue.

**Figure 11:**
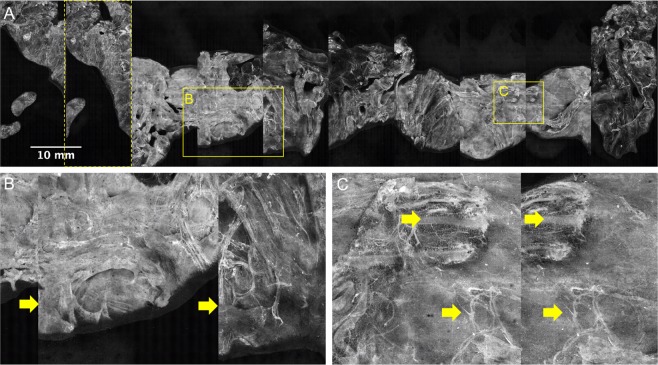
Discontinuous and repeated images in circumferential image of Case 57. The importance of image registration is demonstrated in the image. One panel is mostly repeated, shown by the leftmost dashed yellow box in (**A**). In (**B**), arrows indicate where some adjacent panels are not properly aligned in the vertical direction likely due to longitudinal translation of the prostate along the mounting rod, leading to a discontinuous image. In (**C**) arrows indicate structures that are imaged twice on adjacent panels, likely due to slight free rotation of the prostate on the mounting rod.

Figure 11 A again depicts the prostate specimen of Case 57. Note that the 2 leftmost panels of the image in Fig. 11A appear to be near-repeats of one another. This is in all likelihood due to slippage of the wooden rod in the prostatic urethra during the first lifting and rotation step, which resulted in a repeat imaging of some of the tissue surface (about 90% of the first two panels are the same). A similar but less severe artifact is observed in the right lateral area (3^rd^ and 2^nd^ panels from right), where about 10% of the adjacent panels overlap, again likely due to slippage of the prostate on the wooden rod (Fig. 11C).

### APPS enables higher tissue surface area coverage in less time than prior non-automated method

We hypothesized that the APPS method would increase serviceable surface image coverage of the prostate by a more systematic rotational sampling of the prostate circumference, and our theoretical analysis suggested that an improvement of approximately 10% surface coverage would be possible for a prostate of 20% compressibility. To determine the actual surface image coverage of the manual approach, we first measured the area of tissue from within the collected images using a thresholding approach. We then used a 3D scanner to measure the surface area of the prostate, enabling a comparison of imaged surface area and actual surface area. Table 1 contains data on the imaged surface area fraction and elapsed imaging time for 16 cases in which manual prostate handling was used, and the prostate was successfully 3D scanned. From the table, the range of total prostate surface coverage ranged from as low as 18% to as high as 54%, with a mean of 33.27%. This variability likely results from variation in prostate compressibility and also variation induced by the inefficient handling method itself. In these cases, the average imaging time was 43 ± 6 minutes, and the average imaged area was 21.95 ± 5.18 cm^2^, giving an actual imaging throughput including handling time of 0.5 cm^2^/min. Table 1 also contains data from 2 prostates imaged with the APPS system. Although we were not able to 3D scan these prostates, if we compare the average imaged surface area to the average prostate surface area computed from 16 patients, we find that the APPS may enable a serviceable image coverage of 42%, which is close to our anticipated improvement of 10% surface area coverage over the manual method. However, for these samples, the average elapsed imaging time was only 36 min, and the actual imaging throughput including APPS operation was 0.8 cm^2^/min, which is 1.6× faster than manual handling.

**Table 1:** SIM-imaged tissue surface area, total prostate surface area, percent SIM image area coverage, and elapsed time for prostate specimens imaging using the manual and APPS methods.

Method	Case	SIM image area (cm^2^)	Prostate surface area (cm^2^)	Surface coverage of image (%)	Elapsed Time (min)
Manual	21	18.58	96.90	19.17	39
	22	28.63	86.16	33.23	53
	25	12.62	57.20	22.06	39
	27	21.99	64.60	34.04	42
	28	18.84	68.80	27.38	42
	30	25.19	76.48	32.94	46
	31	12.25	65.70	18.65	35
	33	16.80	56.70	29.63	34
	38	24.48	71.64	34.17	43
	40	27.97	64.12	43.62	46
	41	22.36	66.72	33.51	42
	42	25.14	55.21	45.54	43
	43	29.92	54.44	54.96	40
	44	23.20	58.04	39.97	49
	45	21.33	70.64	30.20	58
	Average	21.95 ± 5.18	67.56 ± 11.49	33.27 ± 9.56	43 ± 6
APPS	57	26.63		39.42*	39
	58	30.64		45.35*	32
	Average	28.64 ± 2.01		42.39 ± 2.97*	36 ± 4

### Development of a fiducial marker system for accurate panel registration

**Figure 12:**
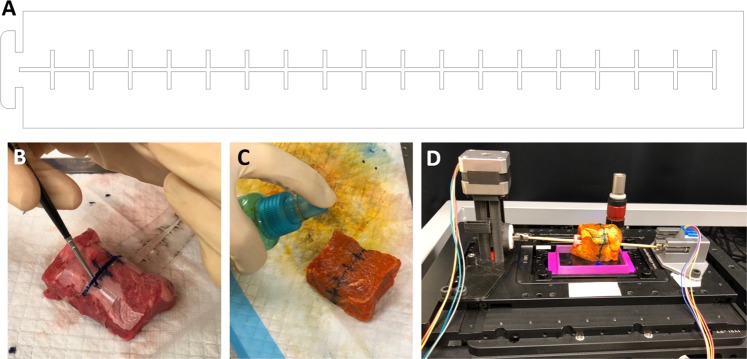
Fiducial markers to aid in image registration. (**A**) The adjustable stencil was laser-cut from a thin piece of clear, flexible plastic. The pattern, as depicted in (**A**) above, consists of one central line and several hash marks spaced evenly apart every 10 mm. (**B**) This stencil is wrapped around the tissue to fit and a thin paintbrush is used to apply histological ink through the stencil gaps. (**C**) The tissue is then stained by spraying with dye and rinsing, and (**D**) mounted in the APPS for imaging. The tissue used in this instance was bovine muscle tissue.

The results underscore the importance of properly securing the prostate onto the rod to prevent slippage, either rotational or translational, from occurring. In addition to ensuring that the prostate is securely mounted on the rod, we also investigated the use of fiducial markers applied to the prostate circumference that could be used to properly orient and stitch adjacent panels, and to detect and correct any slippage if it is to occur. Registration between adjacent panels can be achieved by marking the circumferential surface of the tissue with histological ink prior to imaging. These fiducial markers can be used in post imaging processing to align each panel in the *x* and *y* directions. This can produce a more cohesive or contiguous view of the surgical margin. Stencils were used to ensure uniform fiducial markings. Using a laser cutter, thin sheets of transparent plastic were cut into stencil “bands” that can be wrapped around the prostate and used as a guide when painting on fiducial markings using a small paint brush (Fig. 12).

**Figure 13:**
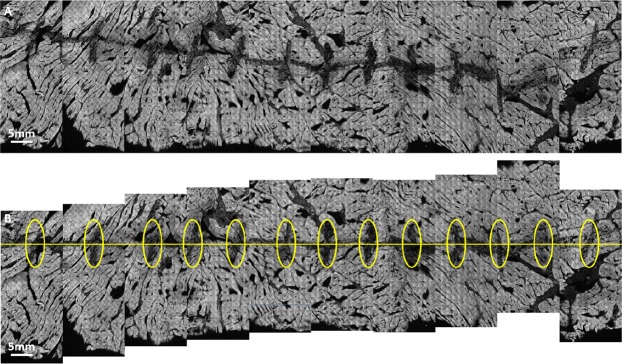
Image registration on fresh bovine muscle tissue. Bovine muscle tissue was marked using the patterned stencil and histological ink, stained with acridine orange, and imaged using SIM coupled with the APPS. (**A**) In the unaligned images, the central line and cross hatches are clearly visible. (**B**) Using the markers as a guide, the panels can be aligned and corrected for rotational or translational slippage.

Figure 13 shows the resulting images of the stencil test before and after manual alignment. The tissue shown is bovine muscle cut into a rough cylinder (d = 5.5 cm, h = 5.2 cm). Using the painted-on fiducial markers as a guide, the images can be aligned along the central line and spread out such that the distance between cross hatches on adjacent panels is equal to the spacing on the stencil. The distance between cross hatches on the stencil used to mark the tissue was 10 mm. This value corresponds well to the measured distance of cross hatches in the image of 10.63 mm. The total SIM surface coverage in this case was 55.76 cm^2^, which corresponds to 62% of the circumferential area of the cylinder (d = 5.5 cm, h = 5.2 cm). The reason for less than 100% of circumferential area coverage was the limited FOV that was captured in this experiment, as the purpose of this test was to image the fiducial markers near the center of the circumference of the cylinder, not to image the entire tissue area.

## Discussion

In our previously-described SIM prostate imaging configuration, we demonstrated in a 36-patient case series that is possible to image the circumferential margin of resected prostate specimens in 49 ± 13 minutes at subcellular resolution, including specimen handling and staining times^10^. The potential for clinical utility of the method was established in that prior work, as 4/5 positive surgical margins (and 4/4 significant positive surgical margins with confirmed viable tumor at the surface) were identified by visual analysis on gigapixel SIM images by blinded pathologist raters who had been trained on a prostate clinical imaging atlas of SIM versus standard H&E. In this work, we aimed to further advance the technology towards clinical translation, by developing a strategy for full automation of sample handling and imaging while simultaneously reducing imaging speed and increasing tissue area coverage. We found that the APPS system does enable improved surface coverage as well as higher image throughput, providing contiguous views of the organ circumference. Importantly, it does not require user intervention during imaging, which is an important objective to promote clinical translation and adoption.

Surface area coverage and intra-operative time are important variables when considering SIM as a valid replacement for frozen section pathology for intraoperative surgical margin assessment of radical prostatectomies. Under-sampling is the main drawback of the current gold-standard pathology method, which can cover at most 0.133% of the total surface area of the prostate (one 4 μm tissue cross-section out of every 3,000 μm thick gross tissue block), and certainly less for intra-operative FSA, where only a few small tissue sites may be sampled. The process to obtain this small percentage of surface area is also time and labor intensive, involving many steps. This discrepancy of systematic histological under-sampling in prostatectomy specimens, even in postoperative permanent pathology, is a provocative issue yet little has been done to improve this process^20,21^. Using a 3D scanner to measure prostate surface area, we found that on average, manual handling and imaging using SIM resulted in tissue surface image areas covering 32.5% of the average prostate, whereas with the new APPS system, this is improved to 42.4% on average, which is a 10% improvement that is in line with our theoretical predictions. Although neither method allows 100% coverage of the prostate, the most important area for intraoperative margin assessment in prostate is arguably the lateral, posterior, and anterior circumferential aspects, which represent areas either containing prominent neurovascular bundles or areas adjacent to important organs (i.e, rectum, bladder). In light of this, SIM imaging combined with APPS enables average area coverages that represent 42% of the average prostate surface area, which is close to the 48% theoretical circumferential area of a cylindrical prostate with 20% compressibility, suggesting that our method is able to sample the vast majority of the prostate circumference. Furthermore, this area coverage represents a 318× improvement over comprehensive gold standard pathology sampling in terms of margin surface coverage percentage, with the latter requiring days of processing. In contrast, we demonstrate that SIM combined with APPS only required on average 36 minutes to acquire a full circumference image, which compares favorably with the typical 20 minute timeframe allotted for a single frozen section intraoperatively, and is significantly less than the 60 minutes that has been reported for intensive intraoperative prostate FSA^22^. Furthermore, this approach would also require fewer personnel resources, since there is no need for gross sectioning, embedding, freezing, cryosectioning, and mounting of multiple separate tissue blocks.

To our knowledge, we present the first circumferential images of the surface of a freshly resected human tumor with subcellular resolution. Thomas *et al*. reported an automated 3-dimensional tumor margin scanner using point probe Raman spectroscopy and white light imaging, that similarly to our system uses tissue rotation to achieve complete tissue coverage in an intraoperative timeframe while reducing user complexity^23^. Both of these systems represent efforts to reduce clinical barriers to adoption for optical tumor margin assessment, based on a recognition that workflow, personnel considerations, time, and cost will be primary drivers for adoption. More studies in a large patient series will be important to validate the utility and clinical acceptability of our approach, and to evaluate its applicability in resection specimens from other organs. A limitation of the present study is that we were not able to 3D scan the particular prostates imaged with the APPS system as we did for the non-APPS imaged prostates; future work will extend these results to obtain a more robust estimate of prostate area surface coverage using the APPS. Additionally, future improvements will develop better methods to securely but safely immobilize the prostate onto the intra-urethral rotating rod, to avoid re-sampling artifacts due to rotational slippage as seen in Fig. 11, while preventing any damage to the specimen. However, our work here demonstrates feasibility and promising first steps toward a completely automated SIM imaging workflow for prostate tumor margin assessment, within accepted intraoperative time limits.
